# Evaluation of Diffusion-weighted MR Imaging as a Technique for Detecting Bone Marrow Edema in Patients with Osteitis Pubis

**DOI:** 10.2463/mrms.mp.2016-0104

**Published:** 2017-02-13

**Authors:** Iclal Erdem Toslak, Bulent Cekic, Aysen Turk, Ali Eraslan, A. Eda Parlak

**Affiliations:** 1 Department of Radiology, Antalya Training and Research Hospital, Varlik Mahallesi, Kazim Karabekir Caddesi 07100 Soguksu, Antalya, Turkey; 2 Department of Sports Medicine, Antalya Training and Research Hospital, Antalya, Turkey

**Keywords:** magnetic resonance imaging, diffusion weighted imaging, bone marrow edema, osteitis pubis, athletic pubalgia

## Abstract

**Purpose:**

Our aims were to determine the feasibility of diffusion-weighted magnetic resonance imaging (DWI) in the detection of bone marrow edema (BME) and explore the apparent diffusion coefficient (ADC) alterations in patients with osteitis pubis (OP).

**Materials and Methods:**

42 consecutive patients clinically suspected to have athletic pubalgia and 31 control subjects were enrolled in the study. All subjects underwent diagnostic focused magnetic resonance imaging (MRI) and DWI at b values of 0 and 600 s/mm^2^. Two radiologists reviewed the images for the presence of active OP. The presence of subchondral BME and contrast enhancement were considered to indicate active OP. ADC values were measured from public bodies of both groups. DWI results were correlated with routine MRI findings. Receiver-operating-characteristic curves were formed. Cut-off values for ADC, sensitivity and specificity values were measured.

**Results:**

36/42 (85%) of the cases had BME/enhancement on routine MRIs and identified as active OP. ADC measurements of the patients were greater than the controls (*P* < 0.05). For the optimal cut-off values DWI showed sensitivity and specificity values of 97.3%, and 90.3%, for the right, and 97.1%, and 96.7% for the left side, respectively (Area under the curve 0.965 and 0.973). Intra-and inter-rater reliability for readers were substantial-perfect for all sessions.

**Conclusion:**

DWI is fast, accurate, and highly reproducible technique for the detection of BME in patients with active OP. It allows distinct bone marrow contrast without the use of gadolinium contrast, increases visual perception of active lesions, gives objective information by quantifying the diffusion coefficients, thus increase diagnostic confidence. We suggest the use of DWI as a cost-effective adjunctive tool for the diagnosis of active OP particularly in early cases and inconclusive diagnostic MRI. Future studies are necessary to determine the utility of DWI to evaluate severity of the disease and treatment response before returning athletes to play.

## Introduction

Athletic pubalgia is a common problem in athletes presenting with exertional pubic or groin pain.^1^ Although this description may include many pathologies, the dominant diagnostic considerations include osteitis pubis (OP), musculotendinous injuries of the rectus abdominus or adductors (adductor dysfunction), and prehernia complex (also termed such as, *sportsman’s hernia, conjoint tendon tear, external oblique tear, and rectus abdominis sheath tears*) that result from posterior inguinal wall deficiency.^1–3^ OP can be a self-limited condition described as inflammation of the symphysis pubis with or without erosion of joint surfaces.^1,4^ Accurate diagnosis and intervention are necessary to reduce symptomatology and to return to optimum play for athletes.^5,6^

Previous studies emphasized the utility of magnetic resonance imaging (MRI) in the diagnosis of OP. They pointed out that identification of bone marrow edema and cortical erosion is highly indicative of osteitis pubis.^1,6–8^ Additionally, the “secondary cleft sign” has been described, with the extension of fluid from primary physiological cleft within the articular disc towards symphyseal enthesis of adductors.^5^ Furthermore, magnetic resonance (MR) has also proven utility for diagnosing OP by excluding alternative diagnoses and by identifying coincident diagnoses including adductor microtear and soft tissue masses.^1,6^

Diffusion-weighted magnetic resonance imaging (DWI) is an MRI technique and the image acquisition of this technique is based upon the ability of stochastic Brownian motion of water molecules to modify signal intensity. Alterations of mobility of water molecules in biological tissues caused by various pathological conditions can be detected with DWI. The term “Apparent Diffusion Coefficient (ADC)” is a quantitative parameter calculated from diffusion weighted images. DWI is used for the evaluation of many musculoskeletal disorders including trauma, tumor and inflammation, and it helps in follow-up of the treatment response by the help of the quantitative information it provides.^9^

The aim of this study was to assess the ability of DWI to detect subchondral bone marrow edema in active OP and to compare to the reliability of other validated MRI sequences like fluid sensitive and contrast-enhanced images thus exploring the utility of the DWI technique related with this issue. To our knowledge, this is the first study concerning the utility of DWI in OP.

## Materials and Methods

This prospective study was conducted in accordance with the 1964 Declaration of Helsinki and was approved by the Institutional Review Board. Written informed consent was obtained from all subjects. Patient records and information were anonymized and de-identified prior to analysis.

### Study population

42 consecutive patients complaining from groin pain for at least six weeks with a possible diagnosis of athletic pubalgia were included in the study. Patients were selected by the department of sports medicine clinic by two physicians and referred to the radiology department for MRI examination. All patients had tenderness over either side of the symphysis pubis region on physical examination. 31 age matched individuals who were undergoing MRI for non-musculoskeletal (MSK) pathology were prospectively enrolled in the study as the control group. All individuals underwent a diagnostic focused pelvic MRI exam and a specific DWI sequence was added to their diagnostic MR imaging protocol. Exclusion criteria were pregnancy, current infections, malignancy, known metabolic diseases, previous interventions to the pubic region, previous pelvic trauma, and general contraindications for MRI (i.e. cardiac pacemakers, neurostimulators, ferromagnetic implants etc). We preferred using a general pelvic evaluation to identify any other components of athletic pubalgia (such as hernia, rectus abdominus strain or adductor strain at the pubic symphysis without OP).

### Magnetic resonance imaging

All patients underwent MR imaging on a 1.5T superconducting eight channel MRI system (Phillips, Achieva) equipped with high speed gradients. Wide field of view (FOV) pelvic MR images in the coronal and axial planes were obtained in the supine position with a high-resolution pelvic phased-array coil. Turbo spin-echo T_1_-weighted (TR/TE: 600/minimum), turbo spin-echo T_2_-weighted (TR/TE: 2100/80), T_2_-weighted spectral presaturation with inversion recovery (SPIR) (TR/TE: 3100/70), T_2_-weighted spectral attenuated inversion recovery (SPAIR) (TR/TE: 5000/70), T_1_-weighted SPIR (TR/TE: 600/minimum) without contrast agents, and T_1_-weighted SPIR (TR/TE: 600/minimum) with gadolinium-based contrast agents (0.1 mmol/kg of body weight) were acquired. All sequences had a slice thickness of 4 mm and number of excitations (nex) of 2.

DWI using single-shot spin-echo echo-planar imaging was performed with diffusion gradient b values of 0 and 600 s/mm^2^. The following DWI parameters were used: FOV 25 (R-L) × 32 (AP) cm; nex: 2; matrix size: 176 × 176; slice thickness: 4 mm; interslice gap, none; TR/TE: 8000/minimum.

### Image interpretation

Before evaluating MR images, identifying information was removed from the images. Readers were blinded to which patients carried a clinical diagnosis of OP. Two radiologists (six and ten years of MSK experience in radiology) evaluated image sets for the presence or absence of bone marrow edema related to active OP, twice from diagnostic MR images, and twice from DWI to assess intra- and inter-observer variability. All evaluations were done at separate sessions with at least two weeks between interpretations.

**Fig 1.**
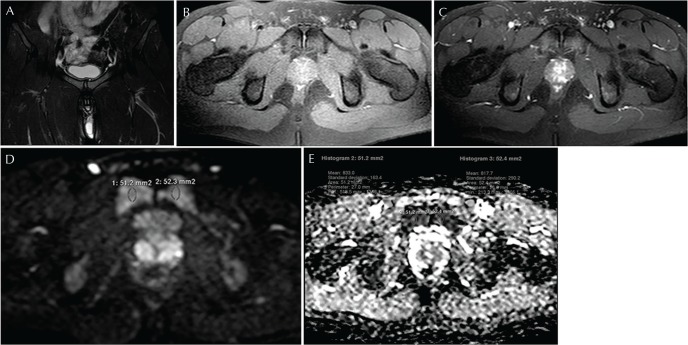
magnetic resonance (MR) images obtained on a 28 year-old soccer player complaining of chronic groin pain for 3 months. (**A**) Coronal T_2_-weigthed spectral presaturation with inversion recovery (SPIR) image shows bone marrow edema on both sides of symphysis pubis joint resulting from osteitis pubis (OP). (**B**) Unenhanced axial T_1_-weighted SPIR image. (**C**) Enhanced T_1_-weighted SPIR image show bone marrow enhancement on both sides of symphysis pubis. (**D**) Axial diffusion-weighted magnetic resonance imaging (DWI) image at b value of 600 s/mm^2^ shows intense high signal on both sides of symphysis pubis joint. (**E**) Apparent diffusion coefficient (ADC) map reveals ADC values of 833 × 10^−6^ mm^2^/s on the right and 818 × 10^−6^ mm^2^/s on left side of symphysis pubis joint which are above the determined threshold value of our study.

Apart from the bone marrow edema, in all individuals, adductor strain, inguinal hernia, rectus abdominus muscle strain and secondary cleft sign were assessed using routine MRI. Subchondral cystic and resorptive changes, joint surface irregularity, and marginal bony spur formation were taken into consideration as structural and chronic changes whereas bone marrow edema was highly suggestive of active OP. Bone marrow edema was defined as hyperintensity on fluid sensitive sequences and hypointensity on T_1_-weighted sequences (Fig. 1A). Active osteitis was indicated by enhancement on T_1_-weighted sequence with fat suppression acquired after administration of paramagnetic contrast gadolinium plus bone marrow edema on either side of the symphysis pubis on fluid sensitive sequences (Fig. 1B and C). The secondary cleft sign was defined as oblique linear high signal intensity extension infero-laterally from the central symphyseal cleft on fluid sensitive MR images as visualized on coronal images.^2,6^ A stress fracture was defined as the decreased signal intensity on T_1_-weighted images and an increased signal intensity on T_2_-weighted and short tau inversion recovery (STIR) images, with or without a low-signal fracture line.^4^ The bone marrow signal intensity of ischial tuberosities was used as the reference for normal bone marrow signal.

After the examination of all images, two radiologists reached a consensus on the differences in readings. Following the consensus reading, the results were combined with the clinical data and a definitive diagnosis of OP was decided by consensus between a radiologist and a sports medicine physician. The diagnosis was considered as positive if at least one side of the patients had bone marrow edema and/or contrast enhancement, and considered as negative if there wasn’t any bone marrow edema or contrast enhancement in pubic symphysis and at least one of the other components of athletic pubalgia was detected which was consistent with the patients’ more symptomatic side.

**Fig 2.**
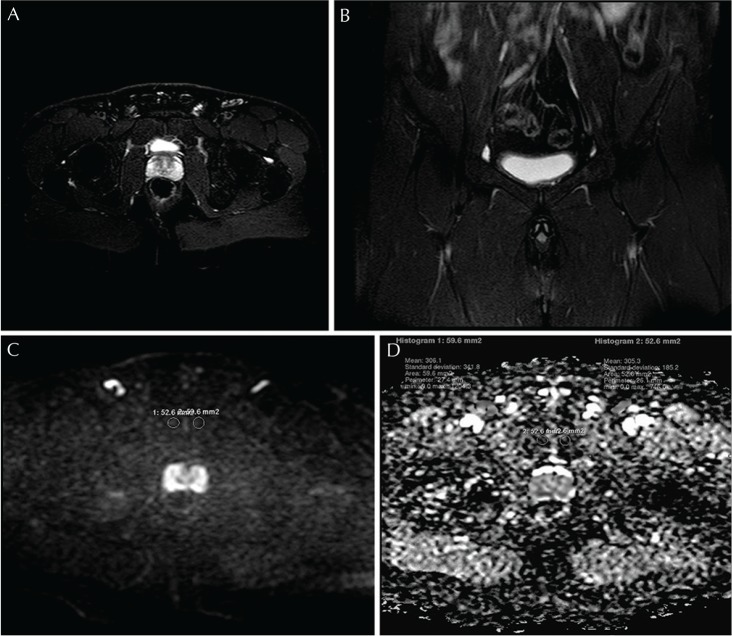
magnetic resonance (MR) images obtained from an asymptomatic 30 year-old man. (**A**) Axial T_2_-weighted spectral pre-saturation inversion recovery (SPIR) image shows normal bone marrow signal in both sides of the joint. (**B**) Coronal T_2_-weighted spectral attenuated inversion recovery (SPAIR) image shows normal bone marrow signal in both sides of the joint. (**C**) Axial diffusion-weighted magnetic resonance imaging (DWI) image at b value of 600 s/mm^2^ shows normal signal in bone marrow. (**D**) apparent diffusion coefficient (ADC) map of the same level. ADC values were 306 × 10^−6^ mm^2^/s on the right and 305 × 10^−6^ mm^2^/s on the left side which were below the determined threshold value of our study.

The diffusion-weighted images were then transferred to a separate workstation (Phillips, Extended MR workspace, 2.6.3.2.HF3, Netherlands). ADC maps were generated. Diffusion weighted images with ADC maps were put on the same screen. A circular region of interest with a value of 40–60 mm^2^; was placed by the radiologists in the subarticular area of pubic bodies for the determination of ADC (Fig. 1D and 1E). Measurements were repeated three times for all of the subjects from each side of the joint. An average value was calculated. ADC values were expressed as square millimeters per second (Fig. 2A–D).

### Statistical methods

Statistical calculations were performed on a personal computer using the SPSS statistical package for Windows 22.0 (SPSS Inc, Chicago, Illinois, USA). Descriptive statistics included frequency, percentage, mean, median, standard deviation (SD) minimum (min), maximum (max). The distribution of categorical variables in groups was compared using the Kolmogorov Simirnov test. Differences between two groups were identified using Mann-Whitney U test. Wilcoxon test was used to assess repeated measurements. The Chi-square test was used to analyze qualitative data. Fischer’s exact test was used instead of Chi-square test for small sample sizes. *P* values lower than .05 were considered statistically significant. Receiver operating characteristic (ROC) curves were formed for DWI sequences, and area under the curve (AUC), sensitivity, and specificity values were calculated to assess the ability of the test to discriminate disease. The definitive diagnosis based on the consensus by a radiologist and sport medicine physician was accepted as the gold standard, and fluid-sensitive and contrast-enhanced T_1_-weighted SPIR images were used as reference standard for the presence of active inflammatory changes. The area under curve values near to 1.0 represented perfect test results; values less than or equal to 0.5 were equivalent or worse results than expected by random chance. The area under curve values had the following interpretation: 0.9 to 0.99 means excellent test, 0.8 to 0.89 means good test, 0.7 to 0.79 means fair test or reasonable test and 0.51 to 0.69 means poor test. The percentage of agreement between the two researchers and the weighted kappa coefficients were calculated with 95% confidence interval (CIs). Kappa agreement was interpreted as follows: <0.01 less than chance agreement, 0.01–0.20 slight agreement, 0.21–0.40 fair agreement, 0.41–0.60 Moderate agreement, 0.61–0.80 Substantial agreement and 0.81–0.99 almost perfect agreement.

## Results

Thirty-six of the 42 clinical patients were diagnosed as active OP on the basis of subchondral bone marrow edema and contrast enhancement in either side of the pubic symphysis and corresponding clinical data. Remaining six patients have been diagnosed as negative for active OP (three had cysts and erosions on pubic symphysis which were consistent with the chronic changes, two had associated unilateral adductor strain, one had unilateral associated rectus muscle strain). Ultimately, our study included 36 patients with active OP (4 female, 32 male; age range between 18 and 66) and 31 control individuals (8 females, 22 males; age range between 18 and 67). Clinical patients with OP consisted of 33 soccer players (91%), 2 tennis players (6%), and a wrestler (3%). Other imaging findings included structural changes on routine diagnostic MR images and they are as follows: Subchondral cystic and resorptive changes and, joint surface irregularity in 16 (44%), associated adductor strain in 17 (47%), rectus abdominus muscle strain in 2 (5%), secondary cleft sign in 16 (44%), and unilateral pubic ramus stress fracture in one (3%) of the patients with active OP. We also detected adductor strain in one (3%) and inguinal hernia in two of the controls (7%).

### Mean ADC values between groups

Baseline marrow sampling of ADC measurements of the ischial tuberosities confirmed similar measurements between patient and control groups. The ADC measurements of bone marrow edema areas of the clinical patients were significantly greater than the control group (*P* < 0.05), regardless of laterality. Table 1 shows ADC values and a comparison of the measurements of patient and control groups.

### ROC analysis of DWI sequence: ability of the test to discriminate active disease

**Fig 3.**
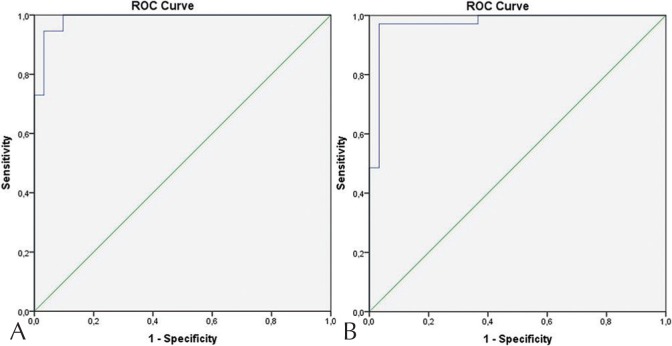
(**A**) Receiver operating characteristics curve of Diffusion-weighted images for the diagnosis of osteitis pubis (OP) on the right side. (**B**) Receiver operating characteristics curve of Diffusion-weighted images for the diagnosis of OP on the left side. ROC, receiver operating characteristic.

For the DWI, ROC curves were formed and AUC values were calculated using the definitive diagnosis based on the consensus by a radiologist and sport medicine physician was as the gold standard (Fig. 3). The AUC values were 0.965 and 0.973 for the right and the left side respectively which means excellent performance for discriminating the disease as compared to routine diagnostic MR images (Table 2). The cut off values for ADC measurements of bone marrow edema for the right and the left sides were found to be 582 × 10^−6^ mm^2^/s and 605 × 10^−6^ mm^2^/s respectively. The sensitivity and specificity values for the optimal cut-off values were 97.3%, and 90.3%, for the right side, and 97.1%, and 96.7% for the left side respectively.

### Intra-and inter-rater reliability

Table 3 shows intra- and inter-rater reliability for routine MRI images, and diffusion-weighted images. Analysis of intra-observer variability for each reader across both reading sessions for both DWI and MR images revealed an almost perfect agreement with kappa (κ) values ranged from 0.851 to 0.974. The inter-rater reliability, was found to be perfect (κ = 0.827) for DWI and substantial (κ = 0.791) for MRI evaluation.

## Discussion

Our results indicate that bone marrow edema causes increased ADC values and DWI enables us both qualitative (increased signal intensity) and quantitative (increased ADC values) information that help discriminate active changes in patients with active OP. To our knowledge, this is the first study evaluates the feasibility of DWI in patients with OP.

OP is a disorder characterized by pain and tenderness over the pubic symphysis.^2^ It is one of the main components of the athletic pubalgia which accounts for 5% of all soccer injuries.^5^ Several etiologies have been proposed including chronic overload or impaction trauma across the pubic bones, sacroiliac joint instability, and excessive shear force on the symphysis pubis. Jumping, twisting, or turning motions during sprinting, cutting, and kicking or rapidly transferring weight from one limb to the other have been postulated as contributory. Soccer, rugby, American football, ice hockey, baseball, Australian Rules football players are most commonly affected.^1,2,5,7^ In the general population, pregnancy, rheumatologic diseases and infection (e.g. urologic infection, osteomyelitis) are predisposing factors for OP.^4^ Accurate diagnosis of OP has clinical importance since the treatment strategies vary considerably from alternative diagnoses of OP.^7^

In the diagnostic imaging protocol of OP, the initial modality is radiography.^3^ Conventional radiographs may show structural changes like cortical marginal irregularity, bony erosions in symphysis pubis (also called *stamp erosions*) which may change into subcortical cysts, osteophytes, and patchy sclerosis. Radiographic bony changes may be late findings after the onset of the symptoms, so alternative imaging modalities may be applied to further characterize.^3,4^ Ultrasound examinations are used to evaluate tendinous pathologies or hernias.^3^ Computerized tomography may be performed to identify more subtle structural bony changes.^4^

Some clinical specialists believe that clinical examination is the mainstay of diagnosis, although MRI does have utility for characterization of findings consistent with OP.^3–7,10,11^ MRI findings depend on the stage of the disease. Among the previously described MRI findings of OP, bone marrow edema reflects the active phase of the disease whereas resorptive changes, subchondral cystic changes, sclerosis, marginal spur formation and joint surface irregularity support chronicity.^1,5–8^ Moreover, Brennan et al. have described the “secondary cleft sign” as a marker of groin injury, that results from a micro tear at the adductor enthesis site, resulting in fluid extending beyond the margins of fibrocartilaginous disk into the enthesis of the adductor and gracilis muscles.^5^

Apart from the commonly applied current practices in neuroradiology and body imaging, DWI has become increasingly popular in musculoskeletal diseases. In some studies regarding the musculoskeletal applications of DWI, some authors concluded that ADC values were increased in traumatic bone marrow edema compared to normal marrow, and others have determined that DWI was useful in differentiating benign and malignant fractures.^12,13^ Additionally, studies have shown that DWI is quite sensitive for detecting bone marrow edema in patients with inflammatory back pain with the added impact of quantifying the degree of the osteitis by ADC measurements. The outcome of those studies promoted the utility of DWI in addition to the standard sequences in the diagnosis of the disease as well as reflecting the inflammatory activity.^14,15^

Diagnostic MRI reveals bone marrow abnormalities in most of OP patients; bone marrow edema is one of the most common findings of OP on MRI. Albers et al.^16^ reported 70% (21 of 30) of OP patients had an abnormal bone marrow signal of the pubic bones whereas Cunningham et al.^1^ found similar findings in 91% (91 of 100), and Verral et al.^2^ reported 72% (76 of 106) bone marrow edema in their study. In the current study, we found that 36 out of 42 clinical patients (85%) had bone marrow edema on either or both sides of the symphysis pubis on diagnostic MRI which was within the acceptable range as the previous literature.^1,2,16^ We included them as in active stages of the disease and building on this, we have found significantly higher ADC values for the bone marrow of the either or both sides of the pubic bodies in those patients compared to the values of the normal bone marrow of the control group on DWI (*P* < 0.001). By using this advantage of DWI, we quantified the bone marrow edema of the patients with active OP, which was an objective data. We found the best cut off value for the disease activation as 582 mm^2^/s on the right and 605 mm^2^/s on the left side using ADC measurements at diffusion gradient b value of 600 s/mm^2^ with sensitivity and specificity values of 97.3%, and 90.3% for the right, and 97.1%, and 96.7%, respectively, which shows DWI is a highly accurate technique in detecting bone marrow edema in patients with active OP. Moreover, both readers could successfully detect bone marrow edema on DWI sequences without any data about the patients’ clinical status or routine MR images with almost perfect intra- and inter-observer agreement proving it as a reliable and highly reproducible method.

To the best of our knowledge, there is not any study in the literature quantifying the bone marrow edema in patients with active OP. Although both STIR and T_2_ fast spin-echo-weighted fat-suppression images are sensitive to detect bone marrow edema in general, the differentiation of bone marrow alterations in these techniques is based on the visual image contrast which is qualitative and subjective. However, DWI, in addition to visual image contrast, has the advantage of quantifying the visually hyperintense lesions which helps increase diagnostic confidence by providing an alternative view of the lesions using a different MR pulse sequence.^17^ Further, it may be helpful for inexperienced readers or when searching for early cases or deciding for inconclusive MRI images in daily clinical practice. As another benefit of DWI, both our study and past studies’ results showed that DWI can detect bone marrow edema as good as contrast enhanced sequences.^15,18^ Further, the acquisition time for DWI sequences is far less than that of the conventional contrast enhanced sequences. Thus, we can conclude that DWI can replace contrast enhanced sequences and help avoid patients from adverse reactions of contrast agents. Moreover, past studies showed that, even though fat suppressed sequences readily identifies posttraumatic bone marrow alterations, the amount of increase in ADC values was higher along with the severity of the injury revealing the necessity of an objective method to determine the severity of injury.^12^

Therefore, on the basis of aforementioned beneficial features of the DWI sequences and based on our study results, we suggest the use of DWI as a cost-effective adjunctive tool in the detection of bone marrow edema in clinically suspected OP patients. Further studies correlating the severity of the disease with ADC values and correlation of clinical findings with follow-up ADC values may lead physicians use of this method in decision making tool for severity of injury and returning athletes to play. Furthermore, we think that with its shorter acquisition time, it can be used as a screening tool for high risk athletes which may allow detect the bone marrow edema in an early period of the disease, and can be used as a preventive tool from more serious outcomes of the disease. Future studies are necessary to show the usefulness of this tool as a screening method.

Our study had some limitations. The first limitation of our study is the lack of comparison with different b values. A broader spectrum of b values would provide more information about the perfusion characteristics of the bone marrow edema areas, although values of 0 and 600 s/mm^2^ have been commonly applied in MSK imaging and were thus applied in this study. One more limitation of our study is that, DWI has relatively low resolution and susceptible to air and bone or soft tissue interfaces. Newer and improved imaging and acquisition techniques on DWI studies for the body parts with less homogenous magnetic field would allow better resolution providing better differentiation of MSK related disorders as well as bone marrow edema in MSK studies. Finally, we lack the recovery information of patients and lack the repeat images over time to determine the pattern of edema resolution. Thus, we couldn’t measure the role of DWI in detecting healed OP and influence of quantitative feature of this technique on the potential alterations in the management of those patients. The long-term follow-up of all patients would provide more information on the recovery process of the disease as well as better understanding the significance of the quantitative nature of the technique.

In conclusion, DWI is a fast, sensitive, reliable and highly reproducible technique in detecting bone marrow edema in active OP. DWI allows distinct bone marrow contrast without the use of gadolinium contrast, increasing visual perception of the active lesions thus avoiding patients from potential adverse effects of contrast agents. Together with the advantage of shorter acquisition time and providing objective data by quantifying the diffusion coefficients, we suggest the use of DWI as a cost-effective adjunctive tool for the diagnosis of active OP which help discriminate bone marrow edema from normal bone and increasing diagnostic confidence, particularly in early cases and inconclusive diagnostic MR images. Future studies to be considered include determining the utility of this quantitative data to evaluate severity of the disease and treatment response before returning athletes to play.

## Figures and Tables

**Table 1.** Comparison of mean ADC values for bone marrow edema areas of clinical patients and normal bone marrow areas of control group obtained from symphysis pubis

	Control group		Patient group		*P*
	
	Mean ± SD (× 10^−6^ mm^2^/s )	Med (min-max) (× 10^−6^ mm^2^/s )	Mean ± SD (× 10^−6^ mm^2^/s )	Med (min-max) (× 10^−6^ mm^2^/s )	
R ADC	420.6 ± 95.4	445 (218–502)	995.3 ± 282.2	970 (584–1810)	0.001
L ADC	405.1 ± 103.6	384 (235–507)	1107.1 ± 346.6	1017 (619–1845)	0.001

Mann-Whitney *U* test/Wilcoxon test. S.D, Standard deviation; ADC, apparent diffusion coefficient.

**Table 2.** Area under the curve values obtained from receiver operating characteristic curve analysis

	Area under the curve	Std. Error^a^	Asymptotic sign.^b^	95% Confidence interval	

				Lower bound	Upper bound
Right ADC	0.965	0.031	0.000	0.904	1.000
Left ADC	0.973	0.020	0.000	0.934	1.000

a Under the nonparametric assumption.

b Null hypothesis, true area = 0.5. ADC, apparent diffusion coefficient.

**Table 3.** Intra- and inter-rater reliability for routine MRI and diffusion-weighted images

Classification system	Reliability analysis		Observed agreement (%)	κ^*^	95% CI^**^ for κ	*P*
DWI	**Intra-observer reliability**						
	Observer A t_1_	Observer A t_2_	0.974	0.942	0.864–1.020	<0.001
	Observer B t_1_	Observer B t_2_	0.927	0.913	0.817–1.009	<0.001
	**Inter-observer reliability**						
	Observer A t_2_	Observer B t_2_	0.921	0.827	0.696–0.958	<0.001
Routine MRI	**Intra-observer reliability**						
	Observer A t_1_	Observer At_2_	0.952	0.912	0.814–1.01	<0.001
	Observer B t_1_	Observer B t_2_	0.915	0.851	0.726–0.976	<0.001
	**Inter-observer reliability**						
	Observer A t_2_	Observer B t_2_	0.903	0.791	0.644–0.938	<0.001

t_1_ represents first reading session and t_2_ represents second reading session.

* κ, Kappa;

** Cl, Confidence interval; DWI, diffusion-weighted magnetic resonance imaging; MRI, magnetic resonance imaging.
